# Assessing Feasibility and Readiness for 3D Printing Integration in Prosthetics and Orthotics: A Cross-Sectional Study

**DOI:** 10.3390/healthcare14193213

**Published:** 2026-09-29

**Authors:** Huthaifa Atallah, Titeana Qufabz, Eman Zmaily, Omar AlTarawneh, Toni Nisula, Bálint Molics

**Affiliations:** 1 Department of Prosthetics and Orthotics, School of Rehabilitation Sciences, The University of Jordan, Amman 11942, Jordan; 2 Department of Biomedical Engineering, Faculty of Engineering and Information Technology, University of Pècs, 7624 Pècs, Hungary; titeanaqofabz@hotmail.com; 3 Department of Applied Pharmaceutical Sciences and Pharmacy Practice, Faculty of Pharmacy, Isra University, Amman 11622, Jordan; eman.zmaily@iu.edu.jo; 4 Health Care Accreditation Council, Amman 11181, Jordan; oaltarawneh@hcac.com.jo; 5 Rehabilitation Team, Metropolia University of Applied Sciences, P.O. Box 4000, Metropolia, 00079 Helsinki, Finland; toni.nisula@metropolia.fi; 6 Department of Sport Physiotherapy, Faculty of Health Sciences, University of Pécs, 7621 Pécs, Hungary; molics.balint@etk.pte.hu

**Keywords:** 3D printing, additive manufacturing, prosthetics and orthotics, Unified Theory of Acceptance and Use of Technology, UTAUT, technology adoption, readiness, feasibility

## Abstract

**Introduction**: Three-dimensional (3D) printing is an emerging technology with the potential to transform prosthetics and orthotics (P&O) education and clinical practice by enabling faster, more customized, and cost-effective device production. However, its adoption of routine P&O practice remains limited, particularly in the Middle East, and little is known about practitioners’ readiness to implement this technology. **Aim**: To evaluate the readiness for and acceptance of integrating 3D printing into P&O practice using the Unified Theory of Acceptance and Use of Technology (UTAUT). **Methodology**: A descriptive cross-sectional study was conducted using a UTAUT-based questionnaire distributed through professional networks and social media. A total of 104 participants, including P&O professionals and final-year P&O students from Jordan, completed the survey. Descriptive statistics and multiple linear regression analyses were performed to identify factors influencing the behavioral intention to adopt 3D printing technology. **Results**: Most participants were familiar with the concept of 3D printing in P&O (62.5%), whereas only 12.5% reported that their workplace currently uses 3D printing for prosthetic or orthotic design or fabrication. Performance Expectancy (3.38 ± 0.66) and Effort Expectancy (3.40 ± 0.70) received moderately positive ratings, while Facilitating Conditions had the lowest mean score (2.58 ± 0.84), indicating limited organizational readiness. Behavioral Intention was moderate (3.02 ± 0.99). Multiple linear regression analysis showed that Performance Expectancy (β = 0.518, *p* < 0.001) was the strongest significant predictor of Behavioral Intention, while Facilitating Conditions (β = 0.215, *p* = 0.008) was also a significant predictor. The model explained 45.5% of the variance in Behavioral Intention (R^2^ = 0.455). The most frequently reported barriers were limited technical expertise, lack of organizational priority, and inadequate access to 3D printing equipment and materials. Participants identified training in computer-aided design (CAD) and improved access to digital manufacturing technologies as key implementation needs. **Conclusions**: P&O professionals and students demonstrated positive attitudes toward 3D printing and a willingness to adopt the technology; however, implementation remains constrained by inadequate infrastructure, limited technical support, insufficient training opportunities, and organizational barriers. Strengthening facilitating conditions through investment in digital infrastructure, curriculum integration, workforce training, and institutional support is likely to accelerate the adoption of 3D printing in P&O education and clinical practice.

## 1. Introduction

The adoption of three-dimensional (3D) printing, also known as additive manufacturing, has increased substantially within healthcare in recent years [1]. In prosthetics and orthotics (P&O), 3D printing offers potential advantages in device customization, manufacturing efficiency, and cost-effectiveness [2,3,4,5]. Its application has expanded across a range of P&O devices, including cranial orthoses, prosthetic sockets, and upper- and lower-limb orthoses [6,7,8,9], with clinical applications reported across different geographical settings, including China [10] and Sierra Leone [10,11].

Despite its considerable potential, the adoption of 3D printing technology in P&O facilities continues to face several barriers worldwide. These challenges include the substantial initial investment required [12], a shortage of personnel with the technical expertise to operate and maintain the technology [13], organizational resistance [14], and concerns regarding the reliability and long-term performance of 3D-printed products [15]. These barriers are particularly pronounced in low- and middle-income countries, where conventional manufacturing methods continue to dominate P&O service delivery [16,17].

Understanding the factors influencing the adoption of new technologies requires an appropriate theoretical framework. Although the Technology Acceptance Model (TAM) primarily explains technology acceptance through perceived usefulness and perceived ease of use, the Unified Theory of Acceptance and Use of Technology (UTAUT) integrates these individual perceptions with social and organizational factors that may influence adoption [18,19,20]. UTAUT was therefore selected for the present study because the implementation of 3D printing in P&O depends not only on users’ perceptions of its usefulness and ease of use but also on organizational support, infrastructure, equipment, technical expertise, and other facilitating conditions. The model includes four principal determinants of technology adoption: Performance Expectancy (PE), Effort Expectancy (EE), Social Influence (SI), and Facilitating Conditions (FC), together with Behavioral Intention (BI) to use the technology. This broader framework was considered particularly appropriate for evaluating readiness for 3D printing within the P&O context.

To the best of our knowledge, no previous study has applied UTAUT to assess readiness for and acceptance of 3D printing technology in the field of P&O. Therefore, this study aimed to evaluate factors influencing the acceptance of 3D printing among P&O clinicians and students in Jordan using the UTAUT framework and to identify barriers, training needs, and organizational factors relevant to its implementation. Based on the UTAUT framework, we hypothesized that higher Performance Expectancy, Effort Expectancy, Social Influence, and Facilitating Conditions would each be positively associated with Behavioral Intention to adopt 3D printing technology.

## 2. Materials and Methods

### 2.1. Study Design

A descriptive cross-sectional questionnaire design was employed. Ethical approval was obtained from the Institutional Review Board (IRB) at the University of Jordan (UoJ) under approval number 500/2025 (approval date: 24 August 2025). All procedures were in accordance with the Declaration of Helsinki.

### 2.2. Questionnaire Development

The questionnaire was adapted from the original UTAUT framework and retained the core constructs of Performance Expectancy, Effort Expectancy, Social Influence, Facilitating Conditions, and Behavioral Intention. The original UTAUT moderating variables were not included in the primary conceptual model, as the study focused on the main adoption-related constructs relevant to 3D printing implementation in P&O.

In addition to the questionnaire constructs, the questionnaire included items addressing participants’ demographic characteristics, awareness and current use of 3D printing in P&O, previous training experiences, perceived training needs, preferred sources of additional information, and perceptions regarding the future implementation of 3D printing technology in clinical practice.

To establish content validity, the initial questionnaire was reviewed by a panel of five experts in P&O, rehabilitation sciences, and healthcare technology from different universities. The experts evaluated the relevance, clarity, comprehensiveness, and appropriateness of the questionnaire items in relation to the study objectives and the UTAUT constructs. Their feedback was incorporated into the questionnaire through revisions to the wording, content, and organization of several items, resulting in the final version used in this study.

In this study, feasibility was conceptualized as perceived implementation feasibility rather than formal technical or economic feasibility. It was assessed through facilitating conditions, perceived resource availability, implementation barriers, and participants’ expectations regarding the future implementation of 3D printing in P&O practice.

### 2.3. Participants and Data Collection

The questionnaire was distributed electronically through LinkedIn, email, WhatsApp, and other social media platforms to ensure broad geographical coverage and clinical representation. The study targeted practicing prosthetists and orthotists, as well as fourth-year undergraduate students in the final month of their BSc Prosthetics and Orthotics programme. Both practicing P&O professionals and final-year P&O students were included to capture perspectives across different stages of professional development. Final-year students were considered relevant because they were approaching entry into clinical practice and represent potential future users of digital P&O technologies. Given the expected differences in clinical experience and exposure to workplace and organizational constraints between students and practicing professionals, participant-group differences were examined separately in the statistical analysis. Data was collected electronically using Google forms. Before the respondents were allowed to participate in the study, electronic informed consent was obtained from all respondents in form of their declaration that they had participated voluntarily, anonymously, and could withdraw from the study at any point without fear. Data were collected between September 2025 and May 2026.

### 2.4. Statistical Analysis

Data were analyzed using IBM SPSS Statistics version 29.0 (IBM Corp., Armonk, NY, USA). Descriptive and inferential statistical analyses were performed. Categorical variables, including demographic characteristics, awareness and use of 3D printing, training status, perceived barriers, training preferences, and support needs, were summarized using frequencies and percentages. Responses to Likert-scale items were coded from 1 (strongly disagree) to 5 (strongly agree), with higher scores indicating more positive perceptions.

The distribution of composite scores was assessed using the Shapiro–Wilk test. As all domain scores significantly deviated from normality, non-parametric statistical tests were applied. The Mann–Whitney U test was used to compare domain scores between two-group variables, including gender, familiarity with 3D printing, current use of 3D printing in the workplace, certified training status, and participant group (clinicians versus students). Comparisons across variables with more than two categories, including age group, educational level, years of professional experience, and facility type, were performed using the Kruskal–Wallis test.

Associations between the UTAUT constructs and Behavioral Intention were examined using Spearman’s rank correlation coefficients. Multiple linear regression analysis was subsequently performed to identify predictors of Behavioral Intention, with Performance Expectancy, Effort Expectancy, Social Influence, and Facilitating Conditions entered as independent variables. Future Outlook was examined descriptively and through bivariate correlation with Behavioral Intention but was not entered into the regression model because it was an exploratory, study-specific construct rather than a component of the core UTAUT model. Associations between categorical variables, including certified training, familiarity with 3D printing, and current workplace use of 3D printing, were examined using the chi-square test.

Because the questionnaire was newly developed for this study, its preliminary psychometric properties were also evaluated. The suitability of the data for factor analysis was assessed using the Kaiser–Meyer–Olkin (KMO) measure of sampling adequacy and Bartlett’s test of sphericity. Because several constructs demonstrated high internal consistency, average inter-item correlations were also calculated for each questionnaire domain to assess the degree of similarity among items and identify potential item redundancy. Statistical significance was set at *p* < 0.05.

## 3. Results

### 3.1. Participant Characteristics

A total of 104 participants completed the questionnaire. Their demographic and professional characteristics are presented in Table 1. Most participants were aged 20–29 years (n = 47, 45.2%) or 30–39 years (n = 36, 34.6%). Females comprised 59.6% of the sample (n = 62), while males accounted for 40.4% (n = 42). The largest participant group consisted of certified prosthetists and orthotists (n = 69, 66.3%), followed by prosthetics and orthotics students (n = 28, 26.9%). More than half of the respondents held a bachelor’s degree (n = 55, 52.9%). Regarding professional experience, 43 participants (41.3%) had 0–5 years of experience, 24 (23.1%) had 11–15 years, and 19 (18.3%) had 16–20 years of experience. Nearly half of the participants (n = 51, 49.0%) were employed in public hospitals or rehabilitation centers, 16 (15.4%) worked in private hospitals or rehabilitation centers, and 37 (35.6%) were affiliated with academic or research institutions, Table 1.

### 3.2. Awareness and Current Use of 3D Printing

Sixty-five participants (62.5%) reported being familiar with the use of 3D printing in prosthetics and orthotics (P&O), whereas 39 (37.5%) indicated no prior familiarity. Despite this level of awareness, only 13 participants (12.5%) reported that their institution currently uses 3D printing for the design or fabrication of prosthetic or orthotic devices, while the remaining 91 participants (87.5%) reported that 3D printing was not currently used in their workplace.

The most frequently reported barriers to implementing 3D printing were a lack of trained personnel or technical expertise (n = 54), low institutional priority (n = 49), limited access to reliable 3D printers or printing materials (n = 46), high initial costs of equipment and software (n = 43), and insufficient institutional support or funding (n = 41). Additional barriers included limited awareness of the benefits and applications of 3D printing (n = 35), satisfaction with conventional manufacturing methods (n = 30), concerns regarding the durability or quality of 3D-printed products (n = 28), resistance to change (n = 25), and the time required for design and printing processes (n = 19), Figure 1.

### 3.3. Reliability of Questionnaire Domains

The internal consistency of the questionnaire was good to excellent across all domains. Cronbach’s alpha coefficients were 0.90 for Performance Expectancy, 0.94 for Effort Expectancy, 0.88 for Social Influence, 0.91 for Facilitating Conditions, and 0.95 for Behavioral Intention. The Future Outlook scale also demonstrated good reliability (Cronbach’s α = 0.81), Table 2.

### 3.4. Perceptions Toward 3D Printing

Among the UTAUT constructs, Effort Expectancy had the highest mean score (3.40 ± 0.70), followed closely by Performance Expectancy (3.38 ± 0.66). Social Influence (3.02 ± 0.74) and Behavioral Intention (3.02 ± 0.99) demonstrated moderate mean scores. Facilitating Conditions had the lowest mean score (2.58 ± 0.84), indicating that participants perceived limited availability of the resources, technical expertise, equipment, materials, and organizational support required to implement 3D printing technology. The Future Outlook score was 2.70 ± 0.99, reflecting moderate but cautious expectations regarding the future integration of 3D printing into prosthetics and orthotics (P&O) practice, Table 3.

### 3.5. Suitability of Data for Factor Analysis

The adoption-related questionnaire items demonstrated good suitability for factor analysis. The Kaiser–Meyer–Olkin (KMO) measure of sampling adequacy was 0.87, indicating good sampling adequacy, and Bartlett’s test of sphericity was statistically significant (χ^2^ = 1969.44, df = 231, *p* < 0.001). These findings indicate that the correlation matrix was suitable for factor analysis; however, KMO and Bartlett’s tests alone do not establish construct validity or confirm the underlying factor structure of the questionnaire. Therefore, these results should be interpreted as a preliminary psychometric assessment rather than evidence of established construct validity. Further validation using exploratory or confirmatory factor analysis in larger and independent samples is warranted.

### 3.6. Correlations with Behavioral Intention

Behavioral Intention was positively associated with all measured constructs. The strongest correlations were observed with Performance Expectancy (Spearman’s rho = 0.55, *p* < 0.001) and Future Outlook (rho = 0.55, *p* < 0.001), followed by Social Influence (rho = 0.51, *p* < 0.001) and Effort Expectancy (rho = 0.36, *p* < 0.001). Facilitating Conditions also demonstrated a weak but statistically significant positive correlation with Behavioral Intention (rho = 0.22, *p* = 0.028), Table 4.

### 3.7. Group Comparisons

Participants who were familiar with 3D printing reported significantly higher Performance Expectancy (3.48 vs. 3.20, *p* = 0.011), Effort Expectancy (3.46 vs. 3.30, *p* = 0.031), Behavioral Intention (3.33 vs. 2.50, *p* < 0.001), and Future Outlook (2.93 vs. 2.31, *p* = 0.004) scores than those who were not familiar with the technology, Table 5.

Participants employed in institutions that currently use 3D printing reported significantly higher Facilitating Conditions scores than those working in institutions without 3D printing (3.37 vs. 2.46, *p* = 0.001). However, no significant differences were observed for Performance Expectancy, Effort Expectancy, Social Influence, Behavioral Intention, or Future Outlook.

Similarly, participants who had received certified training in 3D printing demonstrated significantly higher Facilitating Conditions scores than those without certified training (3.15 vs. 2.47, *p* = 0.003). Certified training, however, was not significantly associated with Behavioral Intention (2.98 vs. 3.03, *p* = 0.967).

Compared with clinicians, students reported significantly higher Performance Expectancy (3.67 vs. 3.27, *p* = 0.001), Behavioral Intention (3.38 vs. 2.89, *p* = 0.050), and Future Outlook (3.46 vs. 2.42, *p* < 0.001). No significant differences between students and clinicians were observed for Effort Expectancy, Social Influence, or Facilitating Conditions.

No statistically significant gender differences were identified across the main UTAUT constructs. Behavioral Intention differed significantly according to educational level (*p* < 0.001), years of professional experience (*p* = 0.018), and institution type (*p* = 0.001). Participants affiliated with academic or research institutions reported the highest Behavioral Intention score (3.49), followed by those working in private hospitals or rehabilitation centers (2.98) and public hospitals or rehabilitation centers (2.69), Table 6.

### 3.8. Predictors of Behavioral Intention

Multiple linear regression analysis (Table 7) demonstrated that the model significantly predicted Behavioral Intention (R^2^ = 0.455, adjusted R^2^ = 0.433; F(4, 99) = 20.63, *p* < 0.001), explaining 45.5% of the variance in Behavioral Intention. Performance Expectancy emerged as the strongest independent predictor (standardized β = 0.518, *p* < 0.001). Facilitating Conditions also significantly predicted Behavioral Intention (standardized β = 0.215, *p* = 0.008). Although Effort Expectancy showed a positive association with Behavioral Intention, it did not reach statistical significance (standardized β = 0.146, *p* = 0.081). Likewise, Social Influence was not a significant predictor (standardized β = 0.036, *p* = 0.700).

### 3.9. Associations Between Training, Familiarity, and Facility Use

Chi-square analysis demonstrated a significant association between certified training in 3D printing and current workplace use of the technology (χ^2^ = 13.68, *p* < 0.001). Among participants who had received certified training, 7 of 16 (43.8%) reported working in institutions that currently use 3D printing, compared with only 6 of 88 (6.8%) participants without certified training. Familiarity with 3D printing was also significantly associated with having received certified training (χ^2^ = 3.86, *p* = 0.049). However, no significant association was observed between familiarity with 3D printing and its current use in participants’ institutions (χ^2^ = 2.12, *p* = 0.146).

### 3.10. Training and Support Needs

Only 16 participants (15.4%) reported having received certified training in 3D printing applications for prosthetics and orthotics (P&O), whereas 88 (84.6%) had not. Despite the limited training exposure, most participants expressed interest in future training opportunities, with 71 (68.3%) indicating “Yes” and an additional 10 (9.6%) responding “Maybe.” The most frequently requested training topics included CAD software for prosthetic and orthotic design modifications (n = 72), 3D scanning techniques (n = 68), material selection (n = 66), an introduction to 3D printing technologies (n = 58), clinical applications and case studies (n = 51), printer operation and maintenance (n = 49), and cost analysis compared with conventional manufacturing methods (n = 42), Table 8.

Participants also identified several key areas of support needed to facilitate the implementation of 3D printing in P&O practice. The most frequently reported needs were access to equipment and materials (n = 70), funding for pilot projects or training (n = 61), technical support and maintenance services (n = 58), clear implementation guidelines or step-by-step instructions (n = 56), mentorship from experienced users (n = 52), institutional or governmental support (n = 42), and collaboration with research institutions or engineering departments (n = 40), Table 9.

### 3.11. Future Outlook

Participants demonstrated generally cautious expectations regarding the future adoption of 3D printing in prosthetics and orthotics (P&O). The perceived feasibility of widespread implementation within the next five years received a mean score of 2.76 ± 1.22, while expectations regarding adoption over the next 5–10 years also remained relatively modest (2.83 ± 1.15), indicating limited confidence in widespread implementation within these timeframes.

The statement that 3D printing would completely replace conventional P&O manufacturing methods received the lowest mean score (2.49 ± 1.18). This finding indicates that participants generally viewed 3D printing as a complementary technology rather than a complete substitute for traditional fabrication methods, reflecting expectations that both manufacturing approaches will continue to coexist in future clinical practice.

## 4. Discussion

To the best of our knowledge, this is the first study to apply the Unified Theory of Acceptance and Use of Technology (UTAUT) to evaluate the readiness for and acceptance of 3D printing technology among prosthetics and orthotics (P&O) practitioners in Jordan. The findings indicate that although participants perceived 3D printing as a useful technology and believed it would be relatively easy to learn and use, its implementation remains constrained by multiple barriers. These include limited infrastructure and resources, organizational challenges, and shortages of trained personnel, all of which may hinder the integration of 3D printing into routine P&O education and clinical practice.

The psychometric assessment demonstrated good suitability of the questionnaire items for factor analysis. The KMO measure of sampling adequacy was 0.87, and Bartlett’s test of sphericity was statistically significant (χ^2^ = 1969.44, df = 231, *p* < 0.001). These findings support the factorability of the questionnaire items and indicate that the correlation matrix was suitable for factor analysis; however, KMO and Bartlett’s tests alone do not establish construct validity. Further psychometric evaluation, including formal assessment of construct validity, in larger and independent samples is warranted.

Evaluation of the UTAUT construct scores revealed a clear distinction between the perceived benefits of 3D printing and the barriers limiting its adoption (Table 3; Figure 1). Performance Expectancy was among the highest-rated constructs, indicating that participants believed 3D printing has the potential to improve clinical outcomes, increase efficiency, and enable greater customization of prosthetic and orthotic devices. These findings are consistent with previous studies demonstrating that 3D-printed P&O devices can achieve comparable or superior fit, reduced manufacturing time, and lower production costs than conventional fabrication methods [1,2,3]. The high Performance Expectancy scores suggest that participants recognize the clinical value of 3D printing, providing a strong foundation for its future adoption in P&O practice.

Effort Expectancy also received a relatively high mean score (3.40 ± 0.70), indicating that participants generally perceived 3D printing technology as relatively easy to learn and use. However, this finding should be interpreted with caution, as only 15.4% of participants had received certified training in 3D printing, and most reported only basic or modest levels of knowledge and practical experience (Table 3). This apparent discrepancy may reflect an optimistic perception of technology’s ease of use rather than actual familiarity with the complete digital workflow, which includes 3D scanning, computer-aided design (CAD), material selection, printing, and post-processing. Alternatively, it may indicate a strong willingness among participants to acquire the knowledge and skills required to implement 3D printing in clinical practice.

Social Influence received a mean score close to the neutral midpoint (3.02 ± 0.74), indicating moderate perceived support for adopting 3D printing technology from colleagues, organizational leadership, and the broader professional environment. Participants also perceived only moderate demand from patients for 3D-printed prosthetic and orthotic devices. This finding is consistent with reports from other low-resource settings, where awareness of advanced P&O technologies among patients and their families remains limited [17]. One possible explanation is that the limited current workplace use of 3D printing (12.5%) has not yet allowed strong professional or organizational norms surrounding its use to develop. The significant bivariate association but non-significant regression coefficient may also indicate overlap between Social Influence and other UTAUT constructs when considered simultaneously. Although Social Influence was positively correlated with Behavioral Intention in the bivariate analysis (Spearman’s rho = 0.51, *p* < 0.001), it was not a significant independent predictor when entered alongside Performance Expectancy, Effort Expectancy, and Facilitating Conditions in the regression model (β = 0.036, *p* = 0.700). This differs from the original UTAUT model, in which Social Influence can play an important role in technology acceptance, particularly under certain conditions and during earlier stages of technology use [16]. One possible explanation is that 3D printing remains at an early stage of implementation within the surveyed Jordanian P&O context, with only 12.5% of participants reporting current workplace use. Consequently, established professional norms, peer expectations, and organizational expectations regarding its use may not yet be sufficiently developed to exert an independent influence on adoption intentions. In addition, the significant bivariate association but non-significant regression coefficient suggests that the contribution of Social Influence may overlap with that of other UTAUT constructs, particularly Performance Expectancy and Facilitating Conditions, when these factors are considered simultaneously. These findings suggest that increasing awareness and promoting the benefits of 3D printing among both healthcare professionals and end users may facilitate its wider adoption.

Facilitating Conditions received the lowest mean score (2.58 ± 0.84), identifying it as an important structural barrier to the adoption of 3D printing in prosthetics and orthotics. Participants consistently reported limited access to 3D printers, printing materials, and technical support (Figure 1). These findings were reinforced by responses to the barrier-related questions, in which high initial costs, limited access to equipment and materials, and insufficient institutional support were among the most frequently reported obstacles. Furthermore, Performance Expectancy emerged as the strongest independent predictor of Behavioral Intention, while Facilitating Conditions also made a significant independent contribution to Behavioral Intention in the regression analysis. These findings indicate that adoption intentions are influenced both by the perceived benefits of 3D printing and by the availability of infrastructure, equipment, materials, technical support, and organizational resources. In contrast, although Effort Expectancy showed a positive association with Behavioral Intention, it did not reach statistical significance (standardized β = 0.146, *p* = 0.081). Similarly, Social Influence was not a significant predictor of Behavioral Intention (standardized β = 0.036, *p* = 0.700). These findings indicate that adoption intentions are influenced both by the perceived benefits of 3D printing and by the availability of infrastructure, equipment, materials, technical support, and organizational resources. In contrast, although Effort Expectancy showed a positive association with Behavioral Intention, it did not reach statistical significance (standardized β = 0.146, *p* = 0.081). Similarly, Social Influence was not a significant predictor of Behavioral Intention (standardized β = 0.036, *p* = 0.700). These findings suggest that perceived benefits and practical organizational readiness may be more influential in shaping technology adoption than perceptions of ease of use or social encouragement. Collectively, the findings highlight the importance of strengthening facilitating conditions while also demonstrating the central role of Performance Expectancy in promoting Behavioral Intention toward 3D printing in P&O practice.

Behavioral Intention demonstrated a moderate mean score, indicating that participants were generally willing to adopt 3D printing technology, although this willingness appeared to depend on the availability of appropriate resources and institutional support. This interpretation is consistent with the regression analysis, which identified Performance Expectancy as the strongest predictor of Behavioral Intention, while Facilitating Conditions also made a significant independent contribution. These findings suggest that participants’ intention to use 3D printing is influenced both by their expectations regarding the benefits and usefulness of the technology and by the availability of the resources and organizational support required for its implementation. Therefore, improving access to equipment, materials, technical support, and training, while also demonstrating the clinical and practical benefits of 3D printing, may strengthen behavioral intention and facilitate the adoption of 3D printing in P&O practice.

Differences between students and practicing clinicians should also be considered when interpreting the findings. Students reported significantly higher Performance Expectancy, Behavioral Intention, and Future Outlook than clinicians, whereas no significant differences were observed for Effort Expectancy, Social Influence, or Facilitating Conditions. These findings suggest that professional status may influence perceptions of 3D printing, particularly expectations regarding its benefits and future adoption. Final-year students may have greater openness to emerging digital technologies, whereas practicing clinicians may evaluate their adoption in the context of existing clinical workflows, resource limitations, and organizational constraints. Therefore, findings derived from the combined sample should be interpreted with consideration of these group differences.

Future Outlook received a relatively low mean score (2.70 ± 0.99), indicating cautious expectations regarding the future integration of 3D printing into routine P&O practice. Although participants recognized the potential benefits of the technology and expressed interest in further training, these positive perceptions did not translate into strong expectations of widespread implementation in the foreseeable future. This cautious outlook may reflect the practical barriers identified in the study, particularly limited access to equipment and materials, insufficient technical expertise, and inadequate organizational support. Accordingly, strategies to promote 3D printing should extend beyond raising awareness of its potential benefits and adopt a phased implementation approach combining targeted training, infrastructure development, technical support, and institutional commitment. Small-scale pilot implementation and supervised practical experience may also help demonstrate feasibility and clinical value and build confidence before wider adoption is pursued.

The low rates of current workplace use (12.5%) and certified training (15.4%) indicate that the adoption of 3D printing technology in the study Jordan remains at an early stage. The principal barriers identified, including limited workforce training, high implementation costs, organizational resistance, concerns regarding product quality, and inadequate infrastructure (Figure 1) are consistent with those reported in the broader healthcare technology adoption literature [21] and previous studies in prosthetics and orthotics [22]. Despite these challenges, participants demonstrated relatively high awareness of 3D printing and a strong interest in further training, suggesting that the P&O workforce is receptive to technological innovation but requires appropriate enabling conditions to support implementation. This interpretation is further supported by the strong demand for training in CAD-based design modification and 3D scanning, as well as requests for improved access to equipment, materials, and technical support. These findings indicate that successful implementation of 3D printing in prosthetics and orthotics requires more than individual willingness to adopt new technologies. Sustainable implementation will depend on coordinated investments in infrastructure, workforce training, institutional support, and access to digital manufacturing technologies. Collectively, these measures could facilitate the integration of 3D printing into routine education and clinical practice, ultimately improving the accessibility, efficiency, and customization of prosthetic and orthotic services.

The limited implementation of 3D printing observed in the present study is likely to reflect interconnected economic, organizational, and regulatory challenges rather than limited acceptance of the technology alone. High initial investment in printers, scanners, software, materials, and maintenance may restrict adoption, particularly in resource-constrained healthcare settings, while the absence of established institutional workflows, adequately trained personnel, technical support, and clear guidance for integrating digitally manufactured devices into routine clinical practice may create additional barriers. These findings have practical implications at multiple levels: healthcare facilities should consider phased implementation through pilot programmes and targeted staff training; universities should strengthen education in 3D scanning, CAD, additive manufacturing, and clinical application; and policymakers and professional bodies should support appropriate standards, regulatory guidance, funding mechanisms, and quality-assurance frameworks for digital P&O services. Future multicenter studies involving larger samples and different countries are needed to validate these findings and determine whether the factors identified in Jordan are transferable to other healthcare and P&O systems.

### Limitations

Several limitations should be considered when interpreting the findings of this study. First, the use of convenience sampling through professional networks and social media may have introduced selection bias, potentially leading to the overrepresentation of younger participants and individuals with a greater interest in digital technologies. Consequently, the findings may not be fully representative of all prosthetics and orthotics (P&O) practitioners in Jordan.

Second, the inclusion of both practicing P&O clinicians and final-year P&O students increased the heterogeneity of the study sample. These groups differ substantially in clinical experience, exposure to organizational constraints, and direct experience with technology implementation in practice, which may have influenced their perceptions of 3D printing. Subgroup analysis demonstrated that students had higher Performance Expectancy, Behavioral Intention, and Future Outlook scores than clinicians, confirming that participant status should be considered when interpreting the combined findings. Although subgroup comparisons were conducted to examine these differences, the relatively small number of students limited more extensive stratified analyses. Future studies with larger samples should consider analyzing students and practicing professionals separately or formally accounting for professional status in multivariable models.

Third, although the questionnaire underwent expert review for content validity, no formal pre-test or pilot study was conducted among the target population before the main data collection. Therefore, item understandability, questionnaire completion time, and preliminary reliability were not assessed in a separate pilot sample. Although the questionnaire demonstrated good to excellent internal consistency and satisfactory preliminary psychometric properties in the study sample, future studies should include pre-testing among representative target participants to further refine item wording and assess questionnaire feasibility before wider implementation. Furthermore, no exploratory or confirmatory factor analysis was performed; therefore, the proposed five-factor structure of the UTAUT-based questionnaire was not formally verified, and its underlying factor structure and construct validity remain to be established. Future studies with larger and independent samples should undertake formal factor analysis to further evaluate the questionnaire’s construct validity.

Future research should employ longitudinal designs to examine changes in technology acceptance and adoption over time and to evaluate the impact of educational programmes, infrastructure development, and organizational interventions on the implementation of 3D printing in prosthetics and orthotics. In addition, qualitative and mixed-methods studies could provide deeper insights into the contextual, organizational, and cultural factors influencing the successful integration of digital manufacturing technologies into P&O education and clinical practice.

Finally, although the sample size was sufficient for the regression analysis, the relatively modest sample size (n = 104) may limit the stability and generalizability of the regression estimates. The observed R^2^ of 0.455 may also decrease when the model is evaluated in independent samples or through cross-validation. Future studies with larger samples are therefore needed to confirm the stability and predictive performance of the model.

## 5. Conclusions

Among the UTAUT constructs, Performance Expectancy emerged as the strongest predictor of Behavioral Intention, while Facilitating Conditions also made a significant independent contribution, highlighting the importance of both perceived clinical value and organizational readiness in technology adoption. The findings demonstrate that participants generally recognized the potential of 3D printing to improve P&O practice and expressed a willingness to adopt the technology. However, implementation remains constrained by limited infrastructure, access to equipment and materials, technical support, and opportunities for formal training. From a scientific perspective, this study extends the application of the UTAUT framework to the emerging field of digital manufacturing in P&O and provides initial evidence on the determinants of 3D printing acceptance and implementation readiness in the Jordanian context.

Among the UTAUT constructs, Performance Expectancy emerged as the strongest independent predictor of Behavioral Intention, while Facilitating Conditions also made a significant independent contribution. These findings highlight the importance of both the perceived benefits of 3D printing and the availability of organizational resources and support. Accordingly, promoting the clinical and practical value of 3D printing, alongside improving infrastructure, technical support, training, and institutional capacity, may facilitate its adoption in P&O education and clinical practice.

Successful integration of 3D printing into routine P&O services will require coordinated efforts from educational institutions, healthcare organizations, policymakers, and professional bodies. Priorities should include integrating digital manufacturing into undergraduate curricula and continuing professional development programmes, expanding practical training in 3D scanning, computer-aided design (CAD), and additive manufacturing, investing in digital infrastructure, and establishing policies that support the implementation of digital technologies within rehabilitation services.

Future research should validate these findings in larger and more diverse samples, including multicenter and international studies, to determine whether the identified adoption factors are consistent across different P&O and healthcare systems.

Overall, the high levels of awareness, positive perceptions, and strong interest in further training observed in this study indicate that the Jordan P&O workforce is receptive to technological innovation. Addressing the identified structural barriers will facilitate the wider adoption of 3D printing and has the potential to improve the efficiency, accessibility, customization, and quality of prosthetic and orthotic services.

## Figures and Tables

**Figure 1:**
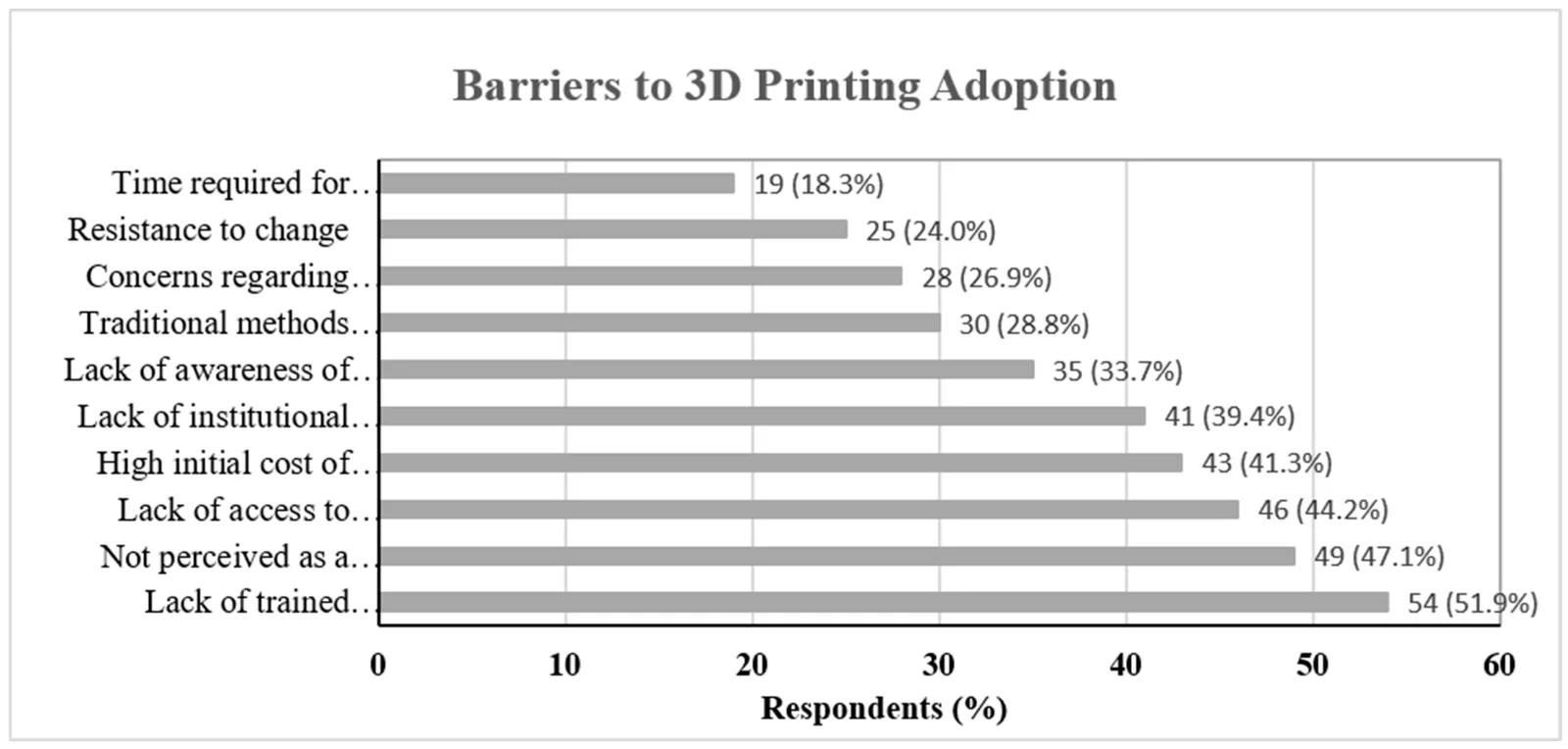
Barriers to 3D Printing Adoption in P&O Practice.

**Table 1:** Demographic and professional characteristics of participants (n = 104).

Variable	Category	n	%
Age	20–29 years	47	45.2
	30–39 years	36	34.6
	40–49 years	18	17.3
	50–59 years	3	2.9
Gender	Female	62	59.6
	Male	42	40.4
Professional role	Certified Prosthetist & Orthotist	69	66.3
	P&O Student	28	26.9
	Certified Prosthetist	3	2.9
	Orthotics Assistant Technician	2	1.9
	Prosthetics Assistant Technician	1	1.0
	Orthotics Technician	1	1.0
Educational level	Bachelor’s Degree	55	52.9
	High School	28	26.9
	Diploma	11	10.6
	Master’s Degree	4	3.8
	Doctorate (PhD)	4	3.8
	Training Certificate	2	1.9
Years of experience	0–5 years	43	41.3
	6–10 years	14	13.5
	11–15 years	24	23.1
	16–20 years	19	18.3
	>20 years	4	3.8
Facility type	Public Hospital/Centre	51	49.0
	Academic/Research Institution	37	35.6
	Private Hospital/Centre	16	15.4

**Table 2:** Reliability analysis of questionnaire domains.

Domain	Number of Items	Cronbach’s α	Interpretation
Performance expectancy	4	0.90	Excellent
Effort expectancy	4	0.94	Excellent
Social influence	4	0.88	Good
Facilitating conditions	6	0.91	Excellent
Behavioral intention	4	0.95	Excellent
Future outlook	4	0.81	Good

**Table 3:** Mean domain scores for perceptions toward 3D printing.

Domain	Mean ± SD
Performance expectancy	3.38 ± 0.66
Effort expectancy	3.40 ± 0.70
Social influence	3.02 ± 0.74
Facilitating conditions	2.58 ± 0.84
Behavioral intention	3.02 ± 0.99
Future outlook	2.70 ± 0.99

**Table 4:** Correlations between main domains and behavioral intentions.

Variable	Spearman’s Rho	*p*-Value
Performance Expectancy	0.55	<0.001
Effort Expectancy	0.36	<0.001
Social Influence	0.51	<0.001
Facilitating Conditions	0.22	0.028
Future Outlook	0.55	<0.001

**Table 5:** Comparison of domain scores according to familiarity with 3D printing.

Domain	Familiar (Mean ± SD)	Not Familiar (Mean ± SD)	*p*-Value
Performance expectancy	3.48 ± 0.66	3.20 ± 0.62	0.011
Effort expectancy	3.46 ± 0.78	3.30 ± 0.54	0.031
Behavioral intention	3.33 ± 0.87	2.50 ± 0.96	<0.001
Future outlook	2.93 ± 0.96	2.31 ± 0.94	0.004

**Table 6:** Comparison of behavioral intentions according to facility type.

Facility Type	Behavioral Intention Mean ± SD
Academic/Research Institution	3.49 ± 0.77
Private Hospital/Centre	2.98 ± 1.01
Public Hospital/Centre	2.69 ± 1.00

**Table 7:** Multiple linear regression analysis predicting behavioral intention.

Predictor	β	*p*-Value
Performance expectancy	0.518	<0.001
Effort expectancy	0.146	0.081
Social influence	0.036	0.700
Facilitating conditions	0.215	0.008

Note. β values are standardized coefficients.

**Table 8:** Most requested training Topics for 3D Printing in P&O.

Training Topic	Frequency
CAD software for modifications	72
3D scanning techniques	68
Material selection	66
Introduction to 3D technologies	58
Clinical use and case studies	51
Printer operation and maintenance	49
Cost analysis versus traditional methods	42

**Table 9:** Most requested Support Needs for Adoption of 3D Printing Technologies.

Support Need	Frequency
Access to equipment and materials	70
Funding for pilot projects/training	61
Technical support and maintenance	58
Clear guides and instructions	56
Mentorship programs	52
Government/institutional support	42
Collaboration with research institutions	40

## Data Availability

The datasets generated and analyzed during the current study are not publicly available due to participant confidentiality and institutional ethical restrictions but are available from the corresponding author on reasonable request.

## Abbreviations

The following abbreviations are used in this manuscript:

UTAUT	Unified Theory of Acceptance and Use of Technology
CAD	Computer-Aided Design
PE	Performance Expectancy
EE	Effort Expectancy
SI	Social Influence
FC	Facilitating Conditions
BI	Behavioral Intention
SPSS	Statistical Package for the Social Sciences (IBM SPSS Statistics)
KMO	Kaiser–Meyer–Olkin (Measure of sampling adequacy)
SD	Standard Deviation
